# Case Report: ^18^F-FDG PET/CT Demonstrating Malignant Spread of a Pulmonary Epithelioid Hemangioendothelioma

**DOI:** 10.3389/fmed.2022.862690

**Published:** 2022-04-04

**Authors:** Ruolin Wu, Xiaotian Xia, Fan Hu, Yajing Zhang, Jingjing Wang, Yong He, Zairong Gao

**Affiliations:** ^1^Department of Nuclear Medicine, Union Hospital, Tongji Medical College, Huazhong University of Science and Technology, Wuhan, China; ^2^Hubei Province Key Laboratory of Molecular Imaging, Wuhan, China; ^3^Department of Nuclear Medicine, The People's Hospital of Honghu, Honghu, China; ^4^Department of Nuclear Medicine, Zhongnan Hospital of Wuhan University, Wuhan, China

**Keywords:** pulmonary epithelioid hemangioendothelioma, ^18^F-FDG, PET/CT, vascular tumor, skeletal metastases

## Abstract

Pulmonary epithelioid hemangioendothelioma (EHE) is a rare vascular malignancy that is typically low-to-intermediate grade. We report a 47-year-old man with a rapidly progressive pulmonary EHE who initially presented with asymptomatic pulmonary nodules. One nodule was mildly hypermetabolic on initial ^18^F-FDG PET/CT. 10 months later, the patient developed severe bone pain and night sweats. Repeat imaging revealed several lung lesions, diffuse pleural thickening, and multiple skeletal metastases with considerably increased tracer uptake. The patient underwent vertebral, pleural, and pulmonary biopsies and a diagnosis of advanced pulmonary EHE was made. His disease progressed despite four courses of antineoplastic therapy, after which he began palliative care. Pulmonary EHE can be aggressive and spread rapidly. Biopsy of hypermetabolic lung lesions using PET/CT guidance might enable early definitive diagnosis.

## Introduction

Epithelioid hemangioendothelioma (EHE) arises from vascular endothelial or pre-endothelial cells and is rarer than other vascular sarcomas (1). The incidence peaks in the fourth to fifth decade and shows a female predominance (2). EHE exhibits unique clinical and molecular characteristics and poses specific therapeutic challenges. EHE can be extremely fast growing and behave like a high-grade sarcoma. To date, no systemic agent has been approved for therapeutic use against EHE. Antineoplastic medications used for sarcoma are often ineffective (3). Rapid diagnosis and treatment as early as possible seem to be crucial to slow disease progression. Pulmonary EHE is uncommon and deserves further investigation as a primary lung cancer.

## Case Description

**Figure 1:**
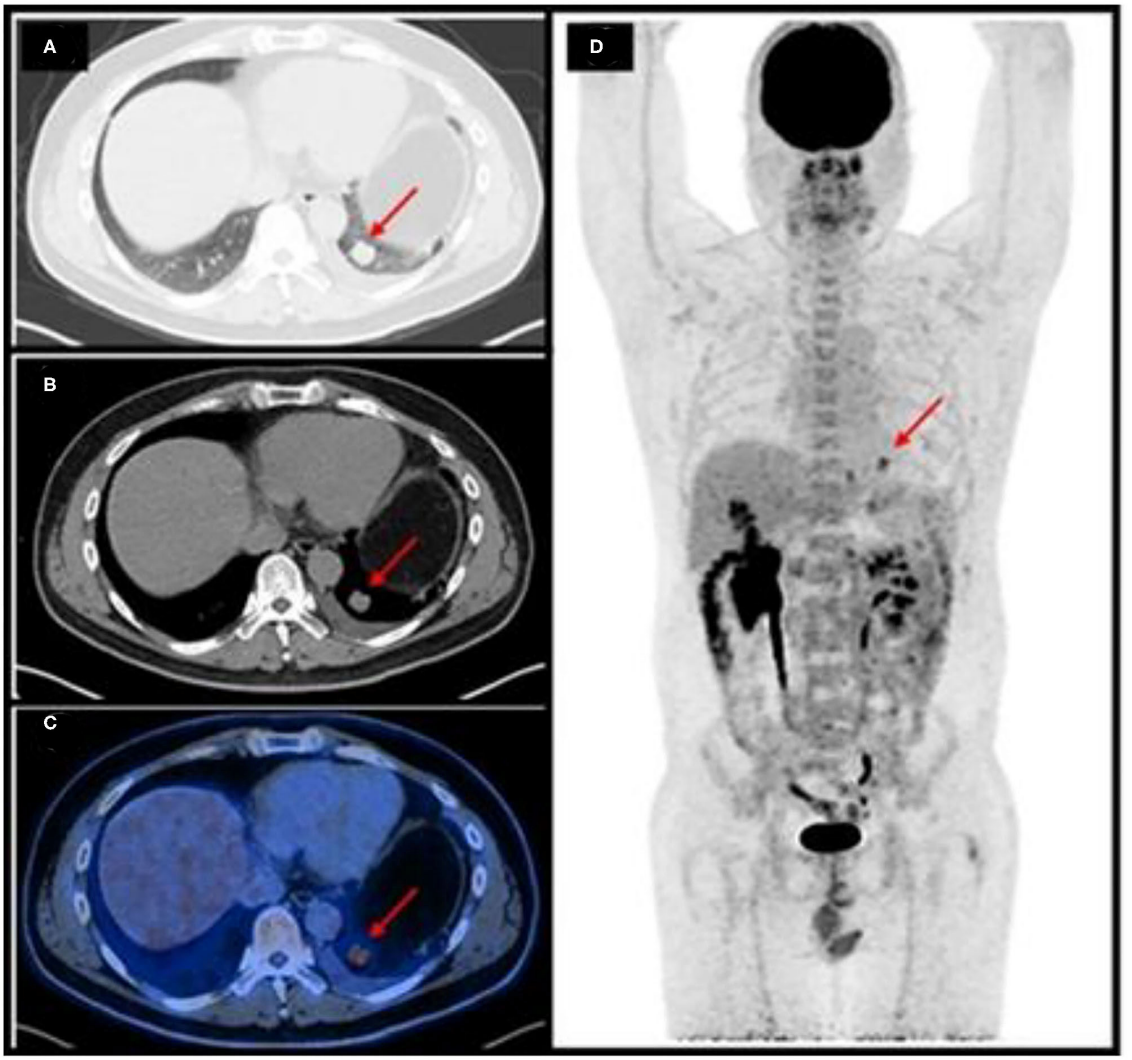
Initial position emission tomography/computed tomography (PET/CT) imaging showed a mildly hypermetabolic lesion in the inferior lobe of the left lung (arrows). **(A,B)** axial CT; **(C)** axial fused PET/CT; **(D)** maximum intensity projection.

**Figure 2:**
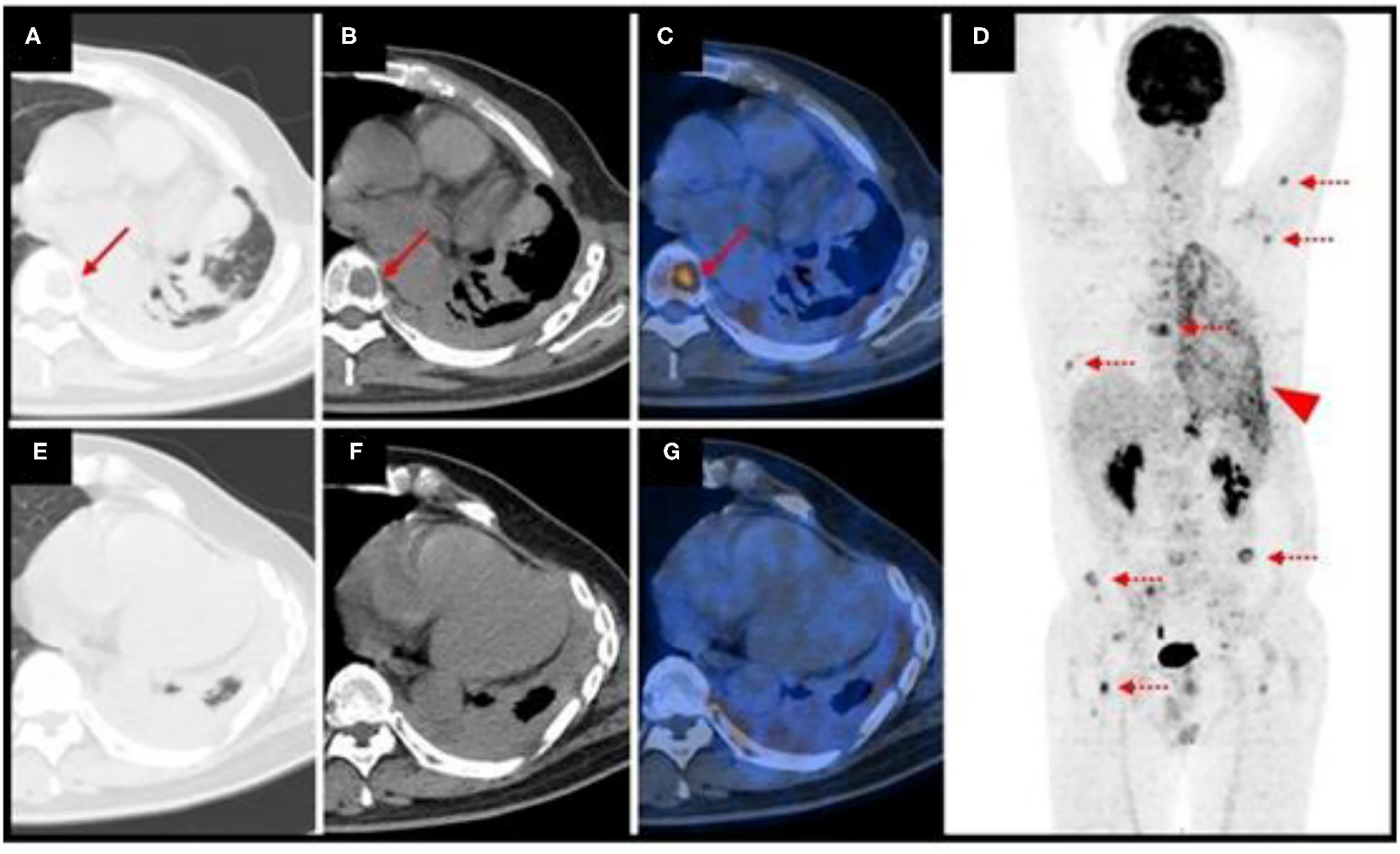
Repeat position emission tomography/computed tomography (PET/CT) imaging showed multiple foci of abnormal activity in the left lung and skeleton. The arrows denote a vertebral body lesion. Dotted arrows indicate multiple bone lesions. The arrowhead indicates intense FDG uptake in the left chest **(A,B,E,F)** axial CT; **(C,G)**, axial fused PET/CT; **(D)** maximum intensity projection.

A 47-year-old man presented with an incidental pulmonary nodule on chest computed tomography (CT). Routine laboratory investigations and tumor markers were within the reference range. The patient had no history of tuberculosis. He underwent ^18^F-fluorodeoxyglucose (FDG) positron emission tomography (PET)/CT, which demonstrated a 1.7 × 2.0 cm mildly hypermetabolic lesion (maximum standardized uptake value, 5.9) in the inferior lobe of the left lung, a small amount of left pleural effusion, and other pulmonary nodules without hypermetabolism (Figure 1). The preliminary diagnosis was inflammatory pulmonary nodules. Nodule biopsy showed caseous necrosis and infiltration of inflammatory cells. We recommended close medical follow*-*up and he was informed that surgical resection may be necessary in the future. Because of his lack of symptoms and the COVID-19 outbreak, the patient asked to be discharged and refused follow-up. 10 months later, he returned complaining of back pain and night sweats for months. SARS-CoV-2 nucleic acid testing was negative. Hematological testing showed a slightly increased inflammatory response. Tumor marker levels were within the reference range except for carbohydrate antigen 125 (36.4 U/mL; reference range, 0–35). CT showed osteolytic lesions in several vertebral bodies. PET/CT showed multiple foci of abnormal activity in the left lung and skeleton as well as pulmonary collapse and pleural effusion. Maximum intensity projection imaging revealed intense FDG uptake in the left chest and multiple bone lesions (Figure 2). The maximum standardized uptake value (SUVmax) is 5.0 for the lung mass shadow, 8.5 for the bone lesions, and 5.1 for the left pleura. The PET/CT studies were used to plan and perform vertebral, pleural, and pulmonary biopsies, which confirmed pulmonary EHE. Based on previously published reports, four courses of antineoplastic therapy were administered. The patient received albumin-bound paclitaxel/nedaplatin. The albumin-bound paclitaxel 260 mg/m^2^ was infused in 30 min, followed by nedaplatin 75 mg/m^2^ over 1.5 h, every 3 weeks. Doses were rounded to the nearest 5 mg. Measurable target lesions were assessed by the Response Evaluation Criteria in Solid Tumors (RECIST) every course. Palliative and supportive care for disease-related symptoms was offered to the patient. Unfortunately, repeat imaging showed disease progression.

## Discussion

In general, EHE is an indolent neoplasm. Reported survival rates range from 6 months to 24 years (1). Over the last two decades, the number of publications regarding EHE has increased more than 5-fold. Although a consensus paper regarding EHE management has been published by the European Society for Medical Oncology (4), treatment has not been standardized and clinical outcomes can be suboptimal, particularly for patients with highly aggressive tumors.

EHE molecular characteristics include WWTR1–CAMTA1 fusion, YAP1–TFE3 fusion, and other rare gene fusions (5–7). Using a conditional EHE mouse model, Seavey et al. demonstrated that WWTR1–CAMTA1 fusion is sufficient to drive EHE formation with exquisite specificity (8). Other studies have examined the role of hormonal stimulation and inflammation in EHE pathogenesis. Pulmonary EHE is radiologically characterized by multiple pulmonary nodules, reticulonodular opacities, diffuse pleural thickening, and parenchymal nodules with pleural invasion (9–12). Although these new studies have greatly enhanced our understanding of the biology of these rare vascular tumors, predictors of degree of malignancy in EHE have not been examined. Elucidating factors involved in malignant progression is a current focus of EHE research. PET/CT, which integrates morphological and metabolic parameters of tumors, may help predict clinical outcome in EHE patients. Preliminary data suggest that patients with lesions exhibiting higher standardized uptake values and those with worse clinical presentations, including anemia, weight loss, fever, fatigue, and tumor-related pain, appear to have significantly worse survival (13, 14). Hypermetabolic lesions associated with diffuse pleural thickening and pleural effusion seem to have a greater risk of progression. Imaging characteristics of serosal involvement and osteolytic alterations, which usually occur in patients with rapidly progressive disease, typically are not seen until after night sweats and bone pain develop.

EHE is often multifocal at the initial presentation. Our patient demonstrated malignant spread from an asymptomatic pulmonary nodule to multiple bones in less than a year. To our knowledge, this has not been previously reported. Because of the potential for rapid progression, EHE should be closely followed.

Several points regarding pulmonary EHE are worthy of discussion. First, it is extremely rare and challenging to diagnose. Its clinical presentation is varied and usually non-specific. Prompt recognition and aggressive patient management are critical. FDG uptake is usually mild-to-moderate. Clinicians and radiologists should be aware of FDG uptake characteristics that can be crucial in diagnosis and prognostication. Second, CT-guided biopsy of pulmonary EHE is associated with limitations. Inadequate imaging of the lesion and inaccurate needle placement may lead to unsuccessful biopsy. This may occur in cases where the lesion is adjacent to inflammation or fibrosis and is difficult to distinguish or resolve. Addition of PET may help. PET/CT is currently used routinely for staging and restaging of various malignancies, owing to its ability to image metabolic activity. This modality allows accurate tumor localization, even when the tumor is surrounded by fibrosis or inflammation. Precise needle placement in the metabolically active area is very important to obtain a representative specimen (15). Using ^18^F-FDG PET/CT guidance for the biopsy procedure may be more helpful and beneficial than routine CT guidance (16). Third, the pathological diagnosis of EHE requires clinical awareness and immunohistochemical staining. Molecular studies should also be performed when it is suspected. Pathologic characteristics of EHE include epithelial cells with abundant eosinophilic cytoplasm and intracytoplasmic vacuolization with a signet ring-like appearance (5). Immunohistochemical staining for the endothelial differentiation markers CD31, ERG, CD34, and FLI-1 can distinguish EHE from other vascular malignancies, as they are consistently expressed in EHE (17, 18). Nuclear expression of CAMTA and TFE can be detected as a result of WWTR1–CAMTA1 and YAP1–TFE3 fusions (5, 6). Disease-related genetic changes that can be detected using next-generation sequencing may be useful in predicting treatment response (19). Although next-generation sequencing was performed in our patient, no significant mutations were identified. Palliative therapy should be considered in patients with advanced disease.

In summary, pulmonary EHE can rapidly progress. PET/CT-guided lung biopsy may enable early diagnosis and treatment. Observation of unifocal EHE is not recommended because of the risk of locoregional or systemic spread. Patients with serosal effusion and marked systemic symptoms tend to have a rapidly progressive course. In these patients, systemic therapy should be considered rather than tumor resection. Future studies should examine survival outcomes in patients with malignant pulmonary EHE.

## Data Availability Statement

The raw data supporting the conclusions of this article will be made available by the authors, without undue reservation.

## Ethics Statement

The studies involving human participants were reviewed and approved by Ethical Committee of Union Hospital, Tongji Medical College. The patients/participants provided their written informed consent to participate in this study.

## Author Contributions

RW, YZ, JW, YH, and ZG obtained and analyzed the clinical data. RW and XX wrote the manuscript. FH designed and constructed the figures. XX and ZG designed the study. All authors contributed to patient care and writing and revising the manuscript and figures.

## Funding

This research was supported by the National Natural Science Foundation of China (Grant Numbers 81801737 and 81771866).

## Conflict of Interest

The authors declare that the research was conducted in the absence of any commercial or financial relationships that could be construed as a potential conflict of interest.

## Publisher's Note

All claims expressed in this article are solely those of the authors and do not necessarily represent those of their affiliated organizations, or those of the publisher, the editors and the reviewers. Any product that may be evaluated in this article, or claim that may be made by its manufacturer, is not guaranteed or endorsed by the publisher.
